# Longitudinal Sleep Patterns and Cognitive Impairment in Older Adults

**DOI:** 10.1001/jamanetworkopen.2023.46006

**Published:** 2023-12-04

**Authors:** Samantha A. Keil, Abigail G. Schindler, Marie X. Wang, Juan Piantino, Lisa C. Silbert, Jonathan E. Elliott, Madeleine L. Werhane, Ronald G. Thomas, Sherry Willis, Miranda M. Lim, Jeffrey J. Iliff

**Affiliations:** 1 VISN 20 Northwest Mental Illness Research, Education and Clinical Center (MIRECC), VA Puget Sound Health Care System, Seattle, Washington; 2 Department of Psychiatry and Behavioral Sciences, University of Washington School of Medicine, Seattle; 3 Department of Radiology, Brain Health Imaging Institute, Weill Cornell Medicine, New York, New York; 4 Geriatric Research Education and Clinical Center (GRECC), VA Puget Sound Health Care System, Seattle, Washington; 5 Gerontology Division, Department of Medicine, University of Washington School of Medicine, Seattle; 6 Now with Seagen, Inc, Bothell, Washington; 7 Department of Pediatrics, Oregon Health & Science University, Portland; 8 Department of Neurology, Oregon Health & Science University, Portland; 9 Neurology Service, VA Portland Health Care System, Portland, Oregon; 10 Oregon Alzheimer’s Disease Research Center, Oregon Health & Science University, Portland; 11 Research Service, VA Portland Health Care System, Portland, Oregon; 12 School of Public Health, University of California, San Diego; 13 Oregon Institute of Occupational Health Sciences, Portland; 14 Department of Neurology, University of Washington School of Medicine, Seattle

## Abstract

**Importance:**

Sleep disturbances and clinical sleep disorders are associated with all-cause dementia and neurodegenerative conditions, but it remains unclear how longitudinal changes in sleep impact the incidence of cognitive impairment.

**Objective:**

To evaluate the association of longitudinal sleep patterns with age-related changes in cognitive function in healthy older adults.

**Design, Setting, and Participants:**

This cross-sectional study is a retrospective longitudinal analyses of the Seattle Longitudinal Study (SLS), which evaluated self-reported sleep duration (1993-2012) and cognitive performance (1997-2020) in older adults. Participants within the SLS were enrolled as part of a community-based cohort from the Group Health Cooperative of Puget Sound and Health Maintenance Organization of Washington between 1956 and 2020. Data analysis was performed from September 2020 to May 2023.

**Main Outcomes and Measures:**

The main outcome for this study was cognitive impairment, as defined by subthreshold performance on both the Mini-Mental State Examination and the Mattis Dementia Rating Scale. Sleep duration was defined by self-report of median nightly sleep duration over the last week and was assessed longitudinally over multiple time points. Median sleep duration, sleep phenotype (short sleep, median ≤7 hours; medium sleep, median = 7 hour; long sleep, median ≥7 hours), change in sleep duration (slope), and variability in sleep duration (SD of median sleep duration, or sleep variability) were evaluated.

**Results:**

Of the participants enrolled in SLS, only 1104 participants who were administered both the Health Behavior Questionnaire and the neuropsychologic battery were included for analysis in this study. A total of 826 individuals (mean [SD] age, 76.3 [11.8] years; 468 women [56.7%]; 217 apolipoprotein E ε4 allele carriers [26.3%]) had complete demographic information and were included in the study. Analysis using a Cox proportional hazard regression model (concordance, 0.76) showed that status as a short sleeper (hazard ratio, 3.67; 95% CI, 1.59-8.50) and higher sleep variability (hazard ratio, 3.06; 95% CI, 1.14-5.49) were significantly associated with the incidence of cognitive impairment.

**Conclusions and Relevance:**

In this community-based longitudinal study of the association between sleep patterns and cognitive performance, the short sleep phenotype was significantly associated with impaired cognitive performance. Furthermore, high sleep variability in longitudinal sleep duration was significantly associated with the incidence of cognitive impairment, highlighting the possibility that instability in sleep duration over long periods of time may impact cognitive decline in older adults.

## Key Points

**Question:**

Are longitudinal patterns of self-reported sleep duration associated with cognitive impairment in older adults?

**Findings:**

In this cross-sectional study of 826 older adults, there was a significant association between cognitive impairment and short sleep duration and longitudinal variability in sleep duration.

**Meaning:**

These findings suggest that longitudinal variability in sleep duration, rather than sleep duration in itself, may be an important factor in the development of cognitive decline in older adults.

## Teaser

This cross-sectional study evaluates the association of longitudinal sleep patterns with age-related changes in cognitive function in healthy older adults.

## Introduction

More than 60 million people globally currently have dementia,^1^ with Alzheimer disease (AD) accounting for an estimated 60% to 80% of these cases.^2,3^ Observational clinical studies demonstrate that many events precede cognitive decline, including amyloid β deposition that appears at least 15 years before the onset of clinical cognitive impairment.^4^ Data from interventional trials^5,6^ suggest that targeting pathogenic processes early in the course of disease may provide the most effective avenue for therapeutic intervention. Identifying the mechanisms preceding cognitive and functional decline may provide approaches to the treatment and prevention decades before dementia onset.

Sleep disruption has long been associated with dementia, with studies^7,8^ indicating that up to 90% of patients experience disrupted sleep before the emergence of cardinal disease symptoms. Although this association was initially believed to reflect the progressive degeneration of sleep-regulatory centers within the brain,^9^ more recent studies suggest that sleep disruption may influence the pathological processes underlying these dementing disorders. Short sleep duration has been associated with increased risk of cognitive impairment in healthy aging adults,^10,11,12,13^ and AD biomarker studies^14,15^ have found that poor sleep quality and short sleep duration are associated with greater AD-related pathological burden. Although these studies suggest a link between chronic sleep disruption and dementia diagnosis, they are limited by their reliance on relatively simple, generally cross-sectional, measures of sleep.

In this study, we evaluate how patterns of longitudinal sleep duration impact age-associated cognitive decline. Using both self-reported sleep duration and a neuropsychological battery regularly collected within the community-based Seattle Longitudinal Study (SLS),^16^ we define the association of longitudinal sleep patterns (duration, change, and variability over time) with the incidence of cognitive impairment in an older population.

## Methods

### Sample

SLS participants were recruited from the Group Health Cooperative of Puget Sound and Health Maintenance Organization of Washington between 1956 and 2020.^16,17,18,19,20^ The present cross-sectional study evaluated a subsample within the SLS that underwent multiple rounds of both a Health Behavior Questionnaire (HBQ) and a neuropsychological battery. Of these individuals, only participants with complete demographic information were included in the analysis. Participants in this study were enrolled under an institutional review board–approved protocol through the University of Washington in accordance with guidelines from the Declaration of Helsinki*.*^21^ All participants provided written informed consent before participation in the Seattle Longitudinal Study. This report follows the Strengthening the Reporting of Observational Studies in Epidemiology (STROBE) reporting guidelines for cross-sectional studies.

Self-reported demographic information included sex (female or male), race and ethnicity (White or other, which combined participant responses of Asian or Pacific Islander, Black, Hispanic, Native American, or any other participant response), years of education (at or below high school, college, graduate school), and apolipoprotein E ε4 (APOE*E4) allele carrier status (carrier vs noncarrier). Race and ethnicity were analyzed in this study as demographic variables, in consideration that chronic sleep disorders may occur at disproportionate rates among certain racial and ethnic groups.

### Participant Testing

The HBQ was provided at 3- to 5-year intervals for a total of 5 times between 1993 and 2012. Item 100 on the SLS HBQ asks, “In the PAST 7 DAYS, how many hours did you usually sleep per 24 hours (not counting naps)?” with Likert scale responses of 1 (≤ 5 hours), 2 (6 hours), 3 (7 hours), 4 (8 hours), and 5 (≥ 9 hours). From these data, we calculated sleep duration (median value of all measurements) and assigned a sleep duration phenotype based on these median values (short sleep, <7 hours; medium sleep, 7 hours; and long sleep, >7 hours). Because of the longitudinal assessments, it was possible to evaluate changes in self-reported sleep over time. We first evaluated whether for each participant’s self-reported sleep durations, the slope of the best-fit line was significantly nonzero. We also measured the overall variability in participant self-reported sleep duration over time as the SD of all measurements, which we defined as sleep variability.

Within the original SLS, a neuropsychological battery was repeated every 5 to 7 years between 1997 and 2019. Within the original study, participants who performed below threshold on select tests were determined to be cognitively impaired through consensus of 2 neuropsychologists.^16,17,18,19,20^ In the present analysis, to best capture distinct changes in global cognitive function, we defined cognitive impairment as performance below threshold on both the Mini-Mental State Examination^22,23,24^ (MMSE; score <26) and Mattis Dementia Rating Scale^25,26,27^ (DRS; score <129). The SLS neuropsychological battery also included the Center for Epidemiologic Studies–Depression Scale (CES-D).^28,29^ In this study, the median CES-D global score was used to define depression as a covariate throughout analysis.

### Statistical Analysis

Data analysis was performed from September 2020 to May 2023. Statistical analyses were completed using Prism statistical software version 9.5 (GraphPad) and Python statistical software version 3.6 (Python Software Foundation). All tests were 2-sided, and *P* < .05 was considered statistically significant. Study characteristics for all demographic characteristics (sex, race and ethnicity, level of education, and APOE*E4 allele carrier status), depression (median CES-D total score), self-reported sleep measures (median sleep duration and sleep phenotype), and cognitive measures (MMSE and DRS) were evaluated stratified by ages younger than 65 years, 65 to 84 years, and 85 years and older. Statistical differences were assessed by χ^2^ test and 1-way analysis of variance (ANOVA) with Šídák multiple comparison test used for post hoc analysis.

To assess the association of sleep parameters with the time to cognitive impairment, we used Cox proportional hazards regression. Hazard ratios (HRs) and 95% CIs were estimated using multivariable adjusted Cox models. Only participants who completed 3 rounds of testing were included in analysis, so that best-fit slopes and SDs of sleep duration values could be calculated. Thus, in this part of the study, participants had between 3 and 5 assessments of self-reported sleep duration). Model 1 controlled for demographic covariates of sex, race and ethnicity, years of education, and APOE*E4 allele carrier status. Model 2 added CES-D median depression score to model 1. The final model 3 added sleep factors: categorical assessment of sleep duration phenotype (short sleeper vs long sleeper), decreasing sleep duration (coded 0 if slope of sleep durations was not significantly decreasing and coded as 1 if slope was significantly decreasing), and sleep variability. All model assumptions were tested and met the criteria. Multicollinearity was assessed by variance inflation factor for covariates, resulting in no variance inflation factor greater than 5. Thus, no covariates were removed from the study.

## Results

### Participant Demographics

**Figure 1.**
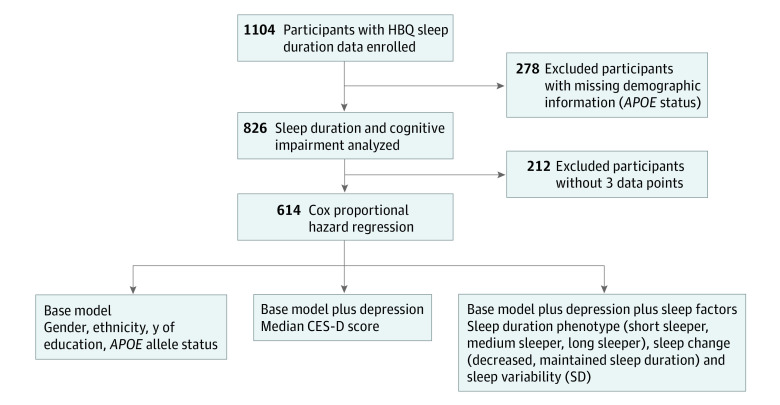
Participant Enrollment Flowchart *APOE* indicates apolipoprotein E; CES-D, Center for Epidemiologic Studies–Depression Scale; HBQ, Health Behavior Questionnaire.

We evaluated 1104 SLS participants and excluded 278 participants lacking demographic data, primarily *APOE* genotype, leaving an initial study sample of 826 participants (mean [SD] age, 76.3 [11.8] years; 468 women [56.7%]; 217 APOE*E4 allele–positive [26.3%]). The flowchart for participant inclusion and exclusion is provided in Figure 1. Table 1 presents the demographic data for sample 1. A significant association was observed between age and years of education, showing that older age groups had lower levels of educational attainment (χ^2^_4_ = 22.96; *P* < .001). No significant difference in APOE*E4 allele status was observed between age groups. Depression was assessed through calculation of median CES-D score for each participant and was compared across age groups. Global CES-D scores were significantly different (*F*_2,823_ = 5.851; *P* = .004; 1-way ANOVA), with 65- to 84-year-old participants reporting lower levels of depression compared with participants younger than 65 years and 85 years or older.

**Table 1.** Baseline Characteristics of Study and Substudy Participants

Characteristic	Participants, No. (%)			Statistical test	*P* value	*P* value for post hoc test^a^
	Aged <65 y (n = 150)	Aged 65-84 y (n = 449)	Aged ≥85 y (n = 227)			
Sample 1 (n = 826)						
Age, mean (SD) [range], y	57.59 (5.71) [40-64]	75.72 (5.35) [65-84]	89.82 (3.61) [85-100]	NA	NA	NA
Sex						
Female	87 (58.0)	248 (55.2)	133 (58.6)	χ^2^	.66	NA
Male	63 (42.0)	201 (44.8)	94 (41.4)			
Race and ethnicity						
White (non-Hispanic)	139 (92.7)	429 (95.5)	219 (96.5)	χ^2^	.22	NA
Other^b^	11 (7.3)	20 (4.5)	8 (3.5)			
Education						
High school or below	9 (6.0)	69 (15.4)	53 (23.3)	χ^2^	<.001	NA
College	74 (49.3)	198 (44.1)	103 (45.4)			
Graduate school	67 (44.7)	182 (40.5)	71 (313)			
Apolipoprotein E ε4 carrier	35 (23.3)	130 (29.0)	52 (22.9)	χ^2^	.16	NA
Depression measure, CES-D, total score, mean (SD) [range]	9.33 (7.16) [0-33]	7.60 (5.84) [0-38]	8.79 (5.68) [0-35]	1-Way ANOVA	.003	.05,^c^ .008^d^
Sleep measures						
Self-reported sleep duration, mean (SD), h	6.65 (0.87)	6.93 (0.87)	7.07 (1.02)	1-Way ANOVA	<.001	.004,^c^ <.001^e^
Sleep phenotype						
Short sleeper: median <7 h	68 (45.3)	145 (32.3)	64 (28.2)	χ^2^	<.001	NA
Medium sleeper: median 7 h	55 (36.7)	168 (37.4)	77 (33.9)			
Long sleeper: median >7 h	27 (18.0)	136 (30.3)	86 (37.9)			
Neuropsychological measures, test score, mean (SD) [range]						
Mini-Mental State Examination Score	29.31 (1.19) [23-30]	28.12 (2.42) [14-30]	26.14 (4.15) [1-30]	1-Way ANOVA	<.001	<.001^c^^,^^d^^,^^e^
Mattis Dementia Rating Scale	141.5 (2.57) [130-144]	139.0 (5.85) [101-144]	131.6 (14.89) [16-144]	1-Way ANOVA	<.001	.01,^c^ <.001^d^^,^^e^
Cognitively impaired	0	20 (4.45)	53 (23.35)	χ^2^	<.001	NA
Sample 2, participants evaluated with Cox proportional hazard regression (n = 614; 143 aged <65 y; 318 aged 65-84 y; 153 aged ≥85 y)						
Age, mean (SD) [range], y	57.66 (5.48) [43-64]	75.07 (5.35) [65-84]	89.77 (3.65) [85-100]	NA	NA	NA
Sex						
Female	83 (58.0)	177 (55.7)	98 (64.1)	χ^2^	.22	NA
Male	60 (42.0)	141 (44.3)	55 (35.9)			
Race and ethnicity						
White (non-Hispanic)	132 (92.4)	306 (96.2)	148 (96.7)	χ^2^	.12	NA
Other^b^	11 (7.6)	12 (3.8)	5 (3.3)			
Education						
High school or below	9 (6.3)	33 (10.4)	28 (18.3)	χ^2^	.009	NA
College	69 (48.3)	135 (42.5)	78 (51.0)			
Graduate school	65 (45.5)	150 (47.2)	47 (30.7)			
Apolipoprotein E ε4 carrier	32 (22.4)	97 (30.5)	35 (22.8)	χ^2^	.09	NA
Depression measure, CES-D, total score, mean (SD) [range]	9.49 (7.25) [0-33]	7.02 (5.59) [0-38]	8.45 (5.68) [0-35]	1-Way ANOVA	<.001	<.001,^c^ .05^d^
Sleep measure						
Self-reported sleep duration, mean (SD), h	6.61 (0.86)	6.82 (0.86)	7.00 (0.98)	1-Way ANOVA	.001	.05,^c^ <.001^e^
Sleep variability (SD)	0.81 (0.38)	0.73 (0.32)	0.72 (0.36)	1-Way ANOVA	.02	.04^c^
Sleep phenotype						
Short sleeper: median <7 h	67 (46.9)	111 (34.9)	44 (28.8)	χ^2^	.002	NA
Medium sleeper: median 7 h	53 (37.1)	130 (40.9)	57 (37.3)			
Long sleeper: median >7 h	23 (16.1)	77 (24.2)	52 (32.0)			
Declining sleep duration	6 (4.2)	25 (7.9)	10 (6.5)	χ^2^	.34	NA
Neuropsychological measures test scores, mean (SD) [range]						
Mini-Mental State Examination Score	29.3 (1.21) [23-30]	28.32 (2.25) [14-30]	26.12 (4.43) [1-30]	1-Way ANOVA	<.001	.002,^c^ <.001^d^^,^^e^
Mattis Dementia Rating Scale	141.5 (2.60) [130-144]	139.8 (4.74) [112-144]	131.9 (16.6) [16-144]	1-Way ANOVA	<.001	<.001^d^^,^^e^
Cognitively impaired	0	11 (3.46)	33 (21.57)	χ^2^	<.001	NA

Abbreviations: ANOVA, analysis of variance; CES-D, Center for Epidemiological Studies Depression Scale; NA, not applicable.

^a^ The Šídák multiple comparison test was used for all post hoc analyses.

^b^ Other race and ethnicity includes Asian or Pacific Islander, Black, Hispanic, Native American, and other participant responses.

^c^ Participants aged 65-84 y vs aged <65 y.

^d^ Participants aged ≥85 y vs aged 65-84 y.

^e^ Participants aged ≥85 y vs aged <65 y.

### Self-Reported Sleep Duration

Although prior studies^10,11,12,13^ have reported shorter sleep durations in older populations, other studies^30,31,32,33^ in have suggested that this association may not be straightforward. Item 100 on the SLS HBQ assesses average nightly sleep duration over the past week (not including naps). From this, we calculated median sleep duration for each participant. We observed a significant age-related difference in sleep duration (*F*_2,823_ = 9.592; *P* < .001; 1-way ANOVA), with longer sleep durations for older participants. When participant median sleep duration phenotype was categorized as short (<7 hours), medium (7 hours), or long (>7 hours), we observed significant differences in the distribution of these phenotypes among age groups (χ^2^_4_ = 20.73; *P* < .001), with an increasing percentage of long sleepers and fewer short and medium sleepers with advancing age.

### Longitudinal Changes in Sleep Duration

Although several studies^10,11,12,13,31^ have sought to define the association of sleep duration assessed at a single time point with cognitive impairment, the repeated assessment of sleep parameters in the present study provides the opportunity to assess how changes in sleep behavior over time contributes to cognitive outcomes. For example, multiple studies^34,35,36,37^ have shown that participants whose self-reported sleep duration declined between 2 assessments at intervals between 3 and 14 years exhibited a higher incidence of cognitive impairment. Because many SLS participants underwent 3 to 5 HBQ assessments, we were able to define generalized patterns of longitudinal sleep duration from these data. Excluding 212 participants from sample 1 who did not have at least 3 HBQ assessments resulted in a subsample of 614 participants (sample 2; Table 1). We evaluated whether each participant exhibited consistent changes in sleep duration over time, whether increasing or declining, by testing if the slope of the best-fit line for sleep durations was significantly positive or negative. Among all participants in sample 2, 41 (6.7%) exhibited a significant decrease in sleep duration, whereas only 4 (0.7%) exhibited a significant increase in sleep duration over time. In our analysis, because of the small number of participants with increasing sleep duration (0.7%), we did not evaluate this group separately. When comparing among the different age groups, we observed no differences in the proportion of participants exhibiting declining sleep duration over time (Table 1).

**Figure 2.**
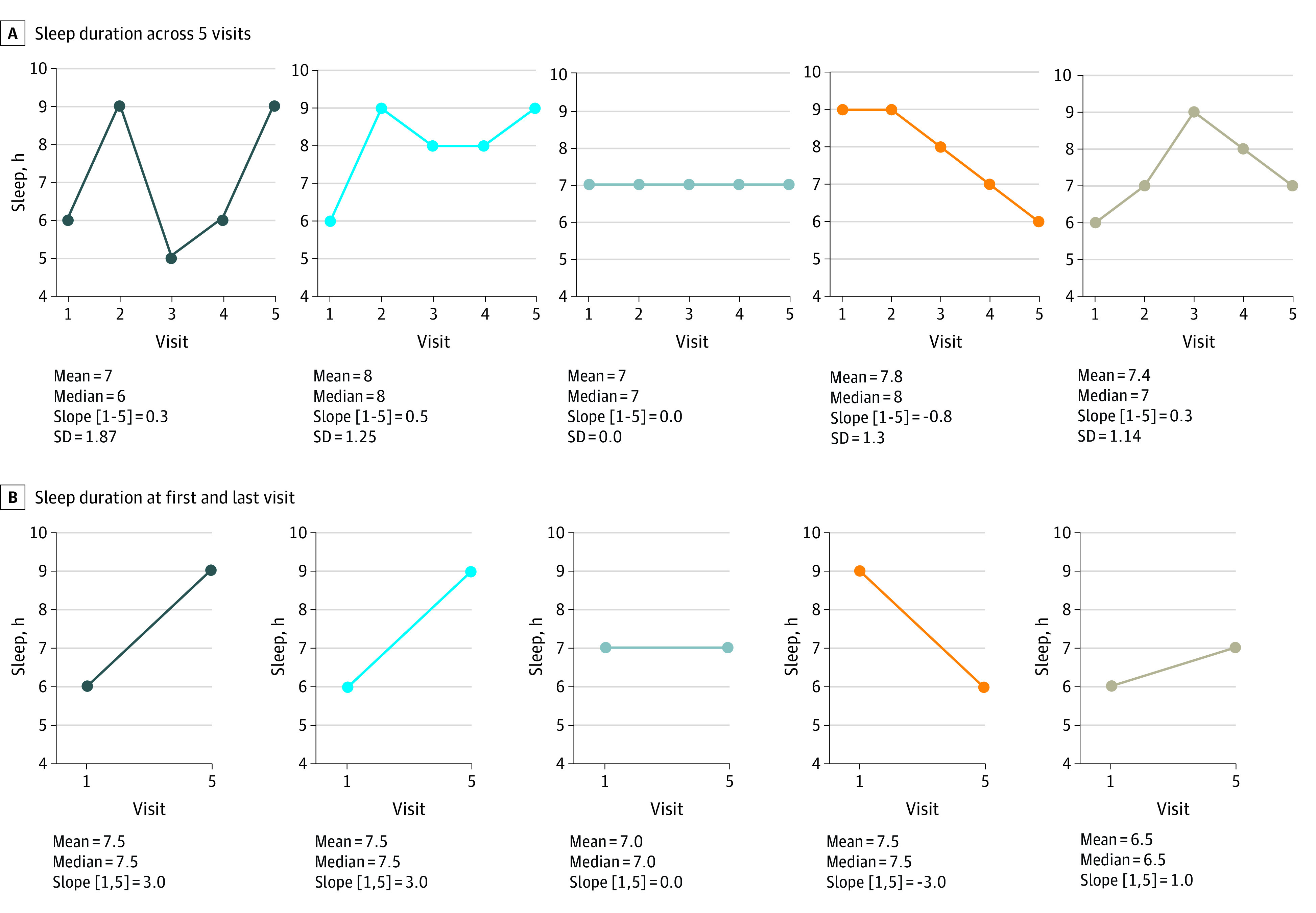
Representative Sleep Patterns Across the Cohort Graphs show sleep duration across 5 visits (A) and differences between the first and last visits (B).

This very low percentage of participants exhibiting a significant change in sleep duration at first seemed inconsistent with prior studies.^34,35,36,37,38^ Figure 2A shows visit-level self-reported sleep duration data from 5 SLS participants who each participated in 5 study visits. It is noteworthy that when 3 data points are censored and only 2 study visits are considered (the first and last), 4 of the 5 participants appear to have clearly increasing or declining sleep durations (Figure 2B). However, when data from all 5 visits are considered, only 1 of the 4 participants exhibits a consistently increasing or declining sleep duration (Figure 2A).

As shown for individual participant values in Figure 2, variability in self-reported sleep duration over several study visits was much more noticeable than were consistent increases or declines in these measures. To capture this feature, we measured sleep variability, defined as the SD of self-reported sleep duration across all participant visits. When compared among age groups, there was a significant association of age with sleep variability (*F*_2,611_ = 3.378; *P* = .02; 1-way ANOVA), with increased age associated with lower sleep variability across longitudinal sleep duration assessments.

### Association of Sleep Parameters With Cognitive Decline

Finally, we evaluated whether these sleep features were associated with cognitive decline using Cox proportional hazards regression. For this analysis, we assessed data up to and including the study visit where participants converted to the status of cognitively impaired (subthreshold performance on MMSE and DRS). For participants who remained cognitively intact for the duration of the study, all data were included.

For the evaluation of cognitive performance within sample 2 based on the MMSE and the DRS (Table 1), as anticipated, we observed higher rates of cognitive impairment with increasing age. Of the 614 participants, 44 (7.2%) exhibited cognitive impairment during their final evaluation, with a higher prevalence of cognitive impairment among participants with older age (χ^2^_2_ = 84.57; *P* < .001).

In model 1, we evaluated the contribution of sex, race and ethnicity, years of education, and APOE*E4 status on the incidence of cognitive impairment with age. Model 1 output is detailed in Table 2 and shows that years of education (HR, 1.16; 95% CI, 1.03-1.28) and possessing 1 or more copies of the APOE*E4 allele (HR, 2.10; 95% CI, 1.11-3.99) were significantly associated with cognitive impairment. We next developed model 2 (Table 2), which added the contribution of depression. Within this model, both APOE*E4 status (HR, 2.08; 95% CI, 1.10-3.97) and years of education (HR, 1.14; 95% CI, 1.03-1.28) were significantly associated with a risk of cognitive impairment. Model 1 and model 2 each had a concordance value of 0.72.

**Table 2.** Cox Proportional Hazard Regression Models Evaluating Longitudinal Associations With Cognitive Impairment for Sample 2 (n = 614)

Model and covariates	HR (95% CI)	*P* value
Model 1, demographic variables (concordance = 0.72)		
Sex	1.08 (0.57-2.04)	.81
Race and ethnicity	0.50 (0.12-2.14)	.35
Years of education	1.16 (1.03-1.28)	.01
Apolipoprotein E ε4 carrier	2.10 (1.11-3.99)	.02
Model 2, demographic variables plus depression (concordance = 0.72)		
Sex	1.08 (0.57-2.04)	.81
Race and ethnicity	0.50 (0.12-2.14)	.35
Years of education	1.14 (1.03-1.28)	.02
Apolipoprotein ε4 carrier	2.09 (1.10-3.97)	.03
Depression (median CES-D total score)	0.98 (0.93-1.04)	.56
Model 3, demographic variables plus depression plus sleep factors (concordance = 0.76)		
Sex	1.31 (0.67-2.56)	.42
Race and ethnicity	0.67 (0.15-2.90)	.59
Years of education	1.25 (1.10-1.42)	<.005
Apolipoprotein E ε4 carrier	2.74 (1.38-5.44)	<.005
Depression (median CES-D total score)	0.97 (0.92-1.03)	.35
Short sleeper	3.67 (1.59-8.50)	<.005
Long sleeper	1.91 (0.83-4.36)	.13
Declining sleep duration	0.50 (0.14-1.79)	.29
Sleep variability (SD)	3.06 (1.14-5.49)	.02

Abbreviations: HR, hazard ratio; CES-D, Center for Epidemiological Studies Depression Scale.

**Figure 3.**
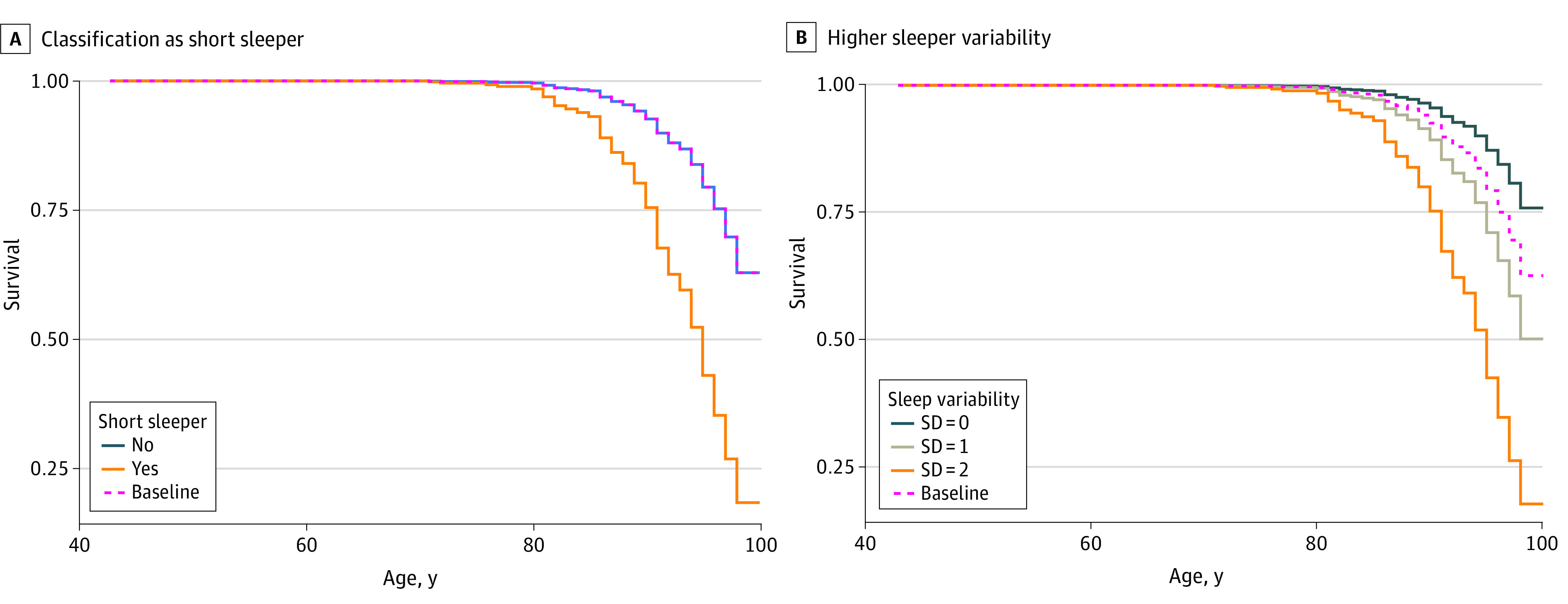
Survival Associated With Sleep Patterns Curves show modeled survival risk curves for patients classified as short sleepers (A) and for those with high sleeping variability (B). Data are not derived from actual patients.

In a series of models based on model 1, we evaluated the association of different sleep parameters, sex, race and ethnicity, years of education, and APOE*E4 status with the incidence of cognitive impairment with age. Within this framework, we observed that neither median sleep duration nor status of declining sleep duration was significantly associated with cognitive impairment. Status as a short sleeper (HR, 2.79; 95% CI, 1.39-6.58) and increasing sleep variability (HR, 2.22; 95% CI, 1.11-5.64) were significantly associated with cognitive impairment with age. We then developed a model (model 3) adding longitudinal sleep parameters, including short sleeper status, status of decreasing sleep duration, and sleep variability, to model 2. Within model 3 (Table 2), several factors were found to be significantly associated with the risk of cognitive impairment, including years of education (HR, 1.25; 95% CI, 1.10-1.42), APOE*E4 status (HR, 2.74; 95% CI, 1.38-5.44), exhibiting a short sleep phenotype (HR, 3.67; 95% CI, 1.59-8.50), and having higher sleep variability (HR, 3.06; 95% CI, 1.14-5.49). Model 3 had a concordance value of 0.76. Using modeled survival curves, Figure 3 shows the associations between short sleep status (Figure 3A) and sleep variability (Figure 3B) and age on remaining cognitively intact among SLS participants. For both short sleeper status and sleep variability, increased risk of cognitive impairment is reflected in the leftward shift in the survival curve.

## Discussion

In this cross-sectional study, analysis of longitudinal measures of self-reported sleep duration within the community-based SLS demonstrated that participants reporting being short sleepers (<7 hours per night) had significantly higher risk of cognitive impairment. These findings are consistent with those of prior studies.^10,11,12,13^ Furthermore, and to our knowledge not previously reported, this study found that higher variability in self-reported sleep duration over the course of decades, rather than a more consistent decline in sleep duration, was significantly associated with cognitive impairment.

The present findings are consistent with previous studies^10,11,12,13^ of healthy aging adults identifying an association of short sleep duration with the risk of cognitive impairment. Several potential mechanisms for the clinical association of chronic sleep disruption include cardiovascular and/or cerebrovascular disease, stroke, metabolic syndrome and/or diabetes, and depression, all of which are known contributors to increased risk of cognitive decline.^39,40,41,42,43,44,45,46,47^ Short sleep duration has been associated with greater amyloid β plaque burden^15^ and faster ventricular expansion,^10^ indicating its potential role in exacerbation of AD-related neurodegenerative processes. Rodent studies demonstrate that sleep-active glymphatic function contributes to the clearance of amyloid β,^48,49^ tau,^50^ and a-synuclein,^51^ whereas acute sleep deprivation slows this clearance.^52^ In addition, recent studies^53,54^ using intrathecal contrast-enhanced magnetic resonance imaging confirmed that solute clearance from the human brain was sleep dependent. Thus, the chronic impairment of sleep-active glymphatic function may underlie the observed clinical association of short sleep duration with cognitive impairment.

We observed that a higher median sleep duration and prevalence of long sleep phenotype was present among older participants in this study. Although this finding was somewhat surprising given the long-established association of aging with shorter sleep,^10,11,12,13^ longer self-reported sleep duration may be observed with increasing age within the cohort for several reasons. It is possible that there is a selection bias within our study, representing an older population with increased sedentary behavior, increased opportunity for sleep with retirement, and changes in comorbidities. Similarly, it is possible that our older participants are overestimating sleep duration in their self-reports, which are known to become increasingly inaccurate with increasing age.^55,56^ Interestingly, although multiple meta-analyses^30,31,32^ have reported that total sleep time does decline with age, this does not extend into the oldest age brackets. In fact, a recent study by Coutrot and colleagues^33^ of 730 187 participants across 63 countries found that shorter sleep durations occurred in persons up to age 50 years compared with young adults. However, these age-related differences plateaued, with an increase in nightly sleep duration being reported in those older than 70 years. Given the advanced age among SLS participants, it is possible that the present study is capturing the sleep patterns in older participants that are often unexplored in studies of healthy aging.

The SLS offers the unique opportunity to examine longitudinal changes in self-reported sleep duration over an extensive follow-up period. With participants providing 3 to 5 HBQ responses over a period of up to 20 years, this allows for the evaluation of both general changes in sleep duration (whether increasing or decreasing) and the intraindividual variability in sleep duration (SD) over time. Previous studies focusing on the association of changes in longitudinal sleep duration with cognition in aging have produced mixed results. Both declining^34,36^ and increasing^35,37,38^ sleep duration have been reported to be associated with cognitive impairment. We believe that one possible cause of these discrepancies stems from most studies quantifying longitudinal changes in sleep duration on the basis of only 2 measurements. As we show for 5 example participants in Figure 2, when longitudinal changes in sleep duration are derived from only 2 assessments, the consistency of change over time is exaggerated. When sampled more frequently, consistent trends in sleep duration become much less prevalent, and variability in sleep duration over the course of years emerges as a key feature of the data. Our observation that sleep variability is significantly associated with cognitive impairment but that general declines in sleep duration are not has important implications for the interpretation of longitudinal sleep data. It is possible that by assessing longitudinal sleep features at only a small number of instances, key biological relationships between changes in sleep and cognitive function are being missed, while spurious associations are arising from undersampled phenomena. Although the present study does not provide clarity on the optimal number or frequency of longitudinal sleep assessments, it does demonstrate that increasing sampling numbers even modestly (such as from 2 to 3-5 instances) provides useful insights regarding sleep variability that would otherwise be missed. It is possible that, in line with the concept of the Nyquist frequency in signal processing, frequent longitudinal assessment of sleep features will be needed to arrive at a clear understanding of the association of age-related changes in sleep with cognitive decline.

The present study reports that increased sleep variability is associated with cognitive impairment. Although this measure reflects a widening range in self-reported sleep duration spanning many years, it remains unclear exactly what this variability reflects. Our analysis suggests that this sleep variability does not simply reflect consistent increases or decreases in sleep duration over time. It is possible that the observed variability reflects changes only in self-reported, and not objective, sleep duration over time. Such changes may originate from cognitive and psychological influences.^57^ The observed variability may be associated with age-related comorbidities in the neurological or psychiatric domains, such as depression, chronic pain, frequent nocturia, or changes in social and behavioral factors,^58^ such as shift work, retirement, or changes in marital status. Future studies will need to define the relationships between long-term variability in self-reported and objective sleep duration and their associations with these cognitive, psychological, social, and behavioral factors.

Our study emphasizes the importance of different experimental approaches when defining longitudinal changes in sleep patterns. Discrepancies in methodological approach, including approaches to assessing sleep duration and quality (eg, self-report, actigraphy, and electroencephalogram), frequency and duration of assessment (single time point assessment, multiday, and long-term ecological assessments), study timescales (from days to years), and synchrony of data collection can each substantially alter study findings. This highlights the need for a more comprehensive evaluation of longitudinal sleep behavior in future studies.

### Limitations

This study has limitations that should be mentioned. The SLS, which was initiated in 1956, aimed to identify the combined impact of aging, birth cohorts, and lifestyle factors on downstream cognitive impairment.^16^ Although the present study did not evaluate birth cohorts, owing to the breadth of study duration, we were able to evaluate both self-reported sleep duration patterns and cognitive performance in 1104 participants assessed over decades. Critically, as a retrospective analysis of SLS data, this study solely relied on self-reported sleep duration as defined by a single question within the HBQ. Although this permits the assessment of longitudinal changes in self-reported sleep patterns, as discussed, the subjective nature of the measure and the infrequent sampling limit the conclusions that we can draw from these data.

In addition, the present study does not assess clinical sleep disorders, such as obstructive sleep apnea,^59,60^ circadian dysregulation,^61^ or insomnia,^62^ which have previously been associated with cognitive impairment. These conditions often exist years before the onset of cognitive decline^7,8^ and may contribute to unstable sleep durations among study participants.

## Conclusions

To our knowledge, this study is the first to demonstrate that variability in longitudinal self-reported sleep duration is associated with cognitive impairment in the community-based SLS sample. It also supports previous findings that a short sleep phenotype is associated with impairment in cognitive performance. These findings suggest that longitudinal variability in sleep duration, in addition to average sleep duration alone, may be important contributors to the development of cognitive decline in older adults. They also argue that understanding the clinical relationships among age, sleep disruption, and cognitive impairment may require the assessment of sleep behavior over longer timescales than is presently common in research practice.

## Notes

Supplement.
Data Sharing Statement
