# Supplementary material for: Longitudinal Sleep Patterns and Cognitive Impairment in Older Adults
Source: JAMA Netw Open. 2023 Dec 4;6(12):e2346006. doi: 10.1001/jamanetworkopen.2023.46006 (PMC10696486; doi:10.1001/jamanetworkopen.2023.46006)
Supplement: Supplement. — Data Sharing Statement [file jamanetwopen-e2346006-s001.pdf]

Data Sharing Statement

Keil. Longitudinal Sleep Patterns and Cognitive Impairment in Older Adults. *JAMA Netw Open*. Published December 04, 2023. doi:10.1001/jamanetworkopen.2023.46006

Data
Data available: Yes
Data types: Deidentified participant data, Data dictionary
How to access data: De-identified patient data are already archived and available through the LONI repository.
When available: With publication

Supporting Documents
Document types: None

Additional Information
Who can access the data: Researchers whose proposed use of the data has been approved
Types of analyses: For any purpose.
Mechanisms of data availability: After approval of proposal.
Any additional restrictions: None.
